# A graftless maxillary sinus lifting approach with simultaneous dental implant placement: a prospective clinical study

**DOI:** 10.1186/s12903-024-03949-9

**Published:** 2024-02-13

**Authors:** Mohammed M. Albadani, Sadam Ahmed Elayah, Mohammed Ali Al-Wesabi, Omar A. Al-Aroomi, Nadia E. Al Qadasy, Hussein Saleh

**Affiliations:** 1 Department of Oral and Maxillofacial Surgery, Jiblah University for Medical and Health Sciences, Ibb, Yemen; 2 Department of Dentistry, Faculty of Medicine and Health Sciences, University of Science and Technology, Aden, Yemen; 3 Faculty of Dentistry, Ibn Al-Nafis University for Medical Sciences, Sana’a, Yemen

**Keywords:** Dental implants, Residual alveolar bone, Maxillary sinus lifting, Simultaneous implantation, Sinus floor elevation, Graftless minimally invasive surgery

## Abstract

**Purpose:**

This study aimed to introduce a graftless sinus lifting approach with simultaneous dental implant placement in the alveolus of the posterior maxilla and compare this approach’s outcomes in freshly extracted sockets versus healed sockets.

**Materials and methods:**

A prospective study was conducted on 60 patients aged between 27 and 59 years old, requiring dental implants in the posterior maxilla, and diagnosed with reduced vertical bone height (30 with freshly extracted sockets (group A) and the remaining 30 with healed sockets (group B). Before the sinus lifting approach, a cone beam computed tomography (CBCT) was taken, followed by another CBCT at least one-year post-sinus lifting (range: 12–36 months). Biological and mechanical complications were assessed, and the primary implant stability was measured using the Implant Stability Quotient (ISQ). Parametric data were analyzed using an independent t-test for intergroup comparisons, with significance set at *P* < 0.05.

**Results:**

No significant differences were found among groups concerning gender, placement side, and follow-up. All dental implants demonstrated high survival rates with no observed biological or mechanical complications. Moreover, the primary implant stability was satisfactory, and there was no statistically significant difference (*P* = 0.38). In terms of new intrasinus bone formation, both groups exhibited satisfactory and successful outcomes, with increased new bone formation in group A. However, there was no statistically significant difference (*P* = 0.26). Regarding the vertical sinus floor elevation without new bone formation, group B showed (0.11 ± 0.64) mm of intrasinus implant height without bone formation, while group A showed an increment of bone formation above the intrasinus implant (0.22 ± 0.33) mm, with no statistically significant difference between both groups (*P* = 0.30).

**Conclusion:**

Our approach proves to be predictable, low-cost, and efficient option for sinus lift procedures, demonstrating high survival rates with acceptable primary implant stability. Moreover, it yields satisfactory outcomes in terms of new intrasinus bone formation, both in freshly extracted and healed sockets. Consequently, our approach holds promise as a reliable procedure for sinus lifting with simultaneous dental implant placement.

## Introduction

The rehabilitation of the posterior maxilla with prosthetic restoration poses a significant challenge due to inadequate bone height and pneumatization of the maxillary sinus [1, 2]. Therefore, a sinus lifting procedure, as described by Tatum [3], reduces the volume of the sinus and facilitates the regeneration of new bone beneath the elevated Schneiderian membrane.

Numerous surgical approaches have been developed to access the sinus cavity and elevate the sinus membrane. There are primarily two types of sinus membrane lifting approaches. The first is known as a two-stage approach, where the maxillary sinus floor is elevated through a lateral window. In this stage, the maxillary sinus membrane is augmented with various bone substitutes, such as autologous, xenogeneic, demineralized, or mineralized allogeneic bone, and alloplasts. Subsequently, the implant is placed after a healing period. The second approach is a one-stage procedure that utilizes either a lateral or transalveolar approach, allowing both membrane lifting and implant placement during the same appointment [4].

While the use of graft materials has shown favorable outcomes in sinus lifting procedures [5, 6], the use of autograft bone presents certain disadvantages. These include the need for an additional surgical site, potential risks of donor site morbidity, postoperative pain, extended operating time, increased expenses, heightened risk of donor site fracture, and a limited graft amount depending on the chosen donor site [7, 8]. Furthermore, allografts, xenografts, and alloplasts encounter various challenges, such as autoimmune rejection, a high risk of disease transmission, residual graft substances, infection, an extended healing period, and their associated high cost [9]. As a response to these challenges, an alternative method was developed, known as the lateral approach without grafting material [2, 10]. While this technique of performing maxillary sinus floor elevation and implant placement simultaneously, without the need for grafting, has proven to be safe and effective with commendable success rates [2], it is not without disadvantages. These include the necessity for a relatively extensive surgical procedure, which demands a steep learning curve and carries the risk of Schneiderian membrane perforation. Additionally, patients may experience a long recovery period and incur higher costs [1]. Nevertheless, the transalveolar approach has been recommended, as it is less invasive and requires less time [11–13]. Consequently, there has been a trend toward prioritizing minimally invasive graftless approaches for sinus lifting, aimed at mitigating the drawbacks associated with lateral sinus lifting procedures [1] and overcoming the disadvantages of sinus lifting with a lateral window. However, it is essential to acknowledge that the availability of minimally invasive graftless approaches might result in a limited variety of sinus lifting techniques. To broaden the surgical indications for minimally invasive graftless sinus lifting techniques, the author (M. Albadani) introduced a novel graftless maxillary sinus lifting approach for simultaneous dental implant placement. The objective of the current clinical study was to describe a graftless sinus lifting approach with simultaneous dental implant placement, inserted either in freshly extracted or healed sockets.

## Materials and methods

### Subjects

A prospective study was conducted on 60 patients aged between 27 and 59 years old, requiring dental implants in the posterior maxilla and diagnosed with reduced vertical bone height during the period from Jan 2021 to Sep 2022. The patients were divided into two groups: 30 patients with freshly extracted sockets (group A), and the other 30 with healed sockets (group B).

The participants were carefully selected based on specific inclusion criteria: (I) patients who needed dental implants in the posterior maxilla and diagnosed with a reduced vertical bone height, at least 5 mm, (II) ages between 27 and 59 years, (III) patients had to be in excellent physical health without any systemic or local illnesses that which can contraindicate the implant or sinus surgery, (IV) Healthy or treated periodontal diseases, (VI) nonsmokers, and (V) no maxillary sinus pathologies. Participants were excluded if they had (I) less than 5 mm of residual bone height, (II) systemic illnesses or medication use that affects bone metabolism, (III) poor oral hygiene or infrequent dental care, (IV) habits like clenching, bruxism, and smoking (V) contraindications for sinus lifting.

The study protocol received ethical approval from the institutional ethics committee at the Department of Oral and Maxillofacial Surgery, Jiblah University for Medical and Health Sciences (JUMHS-050). The study was conducted following the principles outlined in the Declaration of Helsinki. Furthermore, all patients provided written informed consent for their participation in the study.

### Sample size calculation

The sample size calculation was conducted using G*Power 3.0.10 software. A minimum of 25 subjects for each group was determined as necessary. This ensured that, with a significance level set at 0.05, a sample size of 30 participants per group, along with 85% power, would be sufficient to detect any statistically significant differences between the groups. Furthermore, this calculation was based on findings from previous analogous studies [1, 14, 15].

### Surgical protocol

Before surgery, patients rinsed their mouths with 0.12% chlorhexidine gluconate as an antiseptic mouthwash for one minute, followed by the application of local infiltration anesthesia in both the palatal and vestibular aspects. All the sinus lifting operations for both groups were performed by the same experienced implantologist, who used the same dental implants for both groups (diameter of 4.5 and 10 mm length). The implantologist followed the same protocol with the following steps:

#### In freshly extracted socket group

- Perform atraumatic extraction, ensuring thorough curettage and removal of any sharp edges.
- Insert a pilot bur in the bifurcation area, stopping 0.5 mm before reaching the cortical bone of the sinus floor.
- Follow the drilling sequence, ensuring that the final drill used has a diameter 1 mm smaller than the implant.
- Manually insert the implant until it achieves initial stability.
- Use gentle insertion with the wrench, making less than a half turn to establish bi-cortical anchorage.
- Gently tent the sinus membrane to the full length of the implant (implant with a diameter of 4.5 mm and a length of 10 mm, B&B® dental implants, Italy).
- Approximate the gingival edges of the socket using Vicryl 4/0 sutures (Luxcryl 910®, Luxsutures, Italy).
- Use xenograft bone graft material as needed to fill the jumping zone between the implant inside the extracted sockets.
- Place a healing abutment made of PEEK measuring 5 mm to control implant stability and prevent implant sinking into the sinus in case of complications.
- The second stage for prosthesis was carried out after 6 months.

#### In healed socket group

**Fig. 1:**
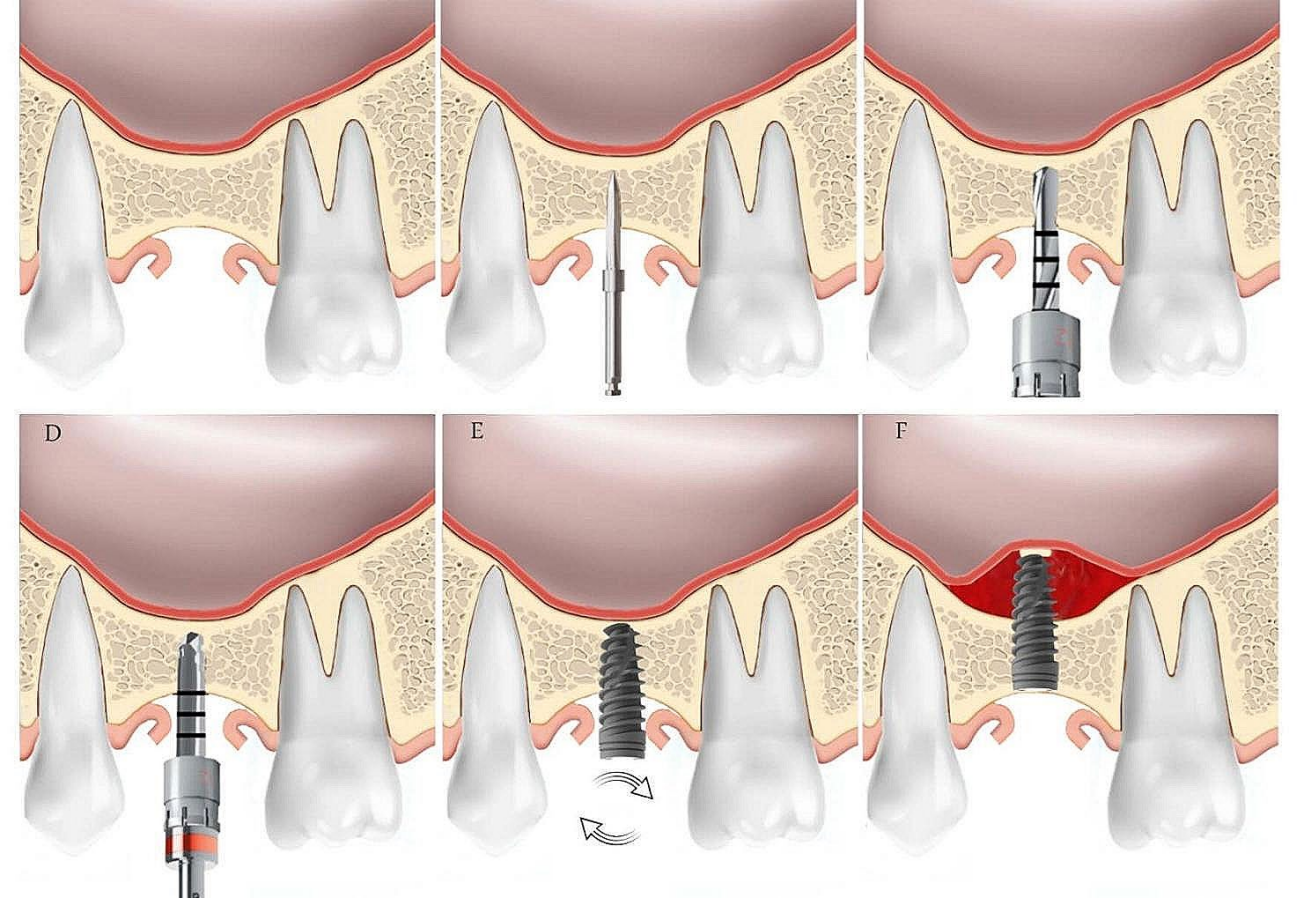
Our approach surgical steps; (**A**) apply a simple mid-crestal incision for mucoperiosteal flap reflection, (**B**) drilling with pilot bur before reaching the cortical bone of the sinus floor by 0.5 mm, (**C & D**) follow the drilling sequence, ensuring the final drill used has a diameter 1 mm smaller than the implant, (**E**) manually insert the implant with clockwise movement, (**F**) Gently tent the sinus membrane to the full length of the implant (implant with a diameter of 4.5 mm and a length of 10 mm, B&B® dental implants, Italy)

- Apply a simple mid-crestal incision for flap design, and then proceed with the same procedure as described above for the group with freshly extracted sockets, with the exception related to dental extraction (Fig. 1).

### Radiologic procedures

A cone beam computed tomography (CBCT) was conducted prior to the surgical procedure (T1) to evaluate the remaining bone dimensions. Subsequently, another CBCT was performed at least one year after the sinus lifting operation (T2). All patients underwent CBCT using the i-CAT® imaging device from Imaging Sciences International, Hatfield, PA, following a standardized protocol. This protocol included a 16.0 cm x 13 cm field of view (FOV), a standardized head position, maximum teeth intercuspation, a horizontal plane parallel to the floor, exposure parameters set at a tube voltage of 120 kV, 18.54 mAs, and a total scan time of 8.9 s. The images were captured at a voxel size of 0.3 mm and stored in Digital Imaging and Communications in Medicine (DICOM) format.

### Assessment of clinical outcomes

According to the evaluation carried out during the 2017 World Workshop on the Classification of Periodontal and Peri-Implant Diseases and Conditions [16], the biological complications, including peri-implantitis and peri-implant mucositis, were assessed. Furthermore, the evaluation of primary implant stability utilized the implant stability quotient (ISQ) [17] with the Osstell ISQ Implant Stability Meter (Integration Diagnostics in Gothenburg, Sweden) [18, 19]. We recorded the mean values of occlusal and buccal ISQ, emphasizing their significance due to the implant’s posterior location and exposure to robust occlusal forces. Moreover, we evaluated mechanical complications, such as implant fracture and failure, following the definitions outlined by the International Team of Implantology [20].

### Assessment of radiologic outcomes

**Fig. 2:**
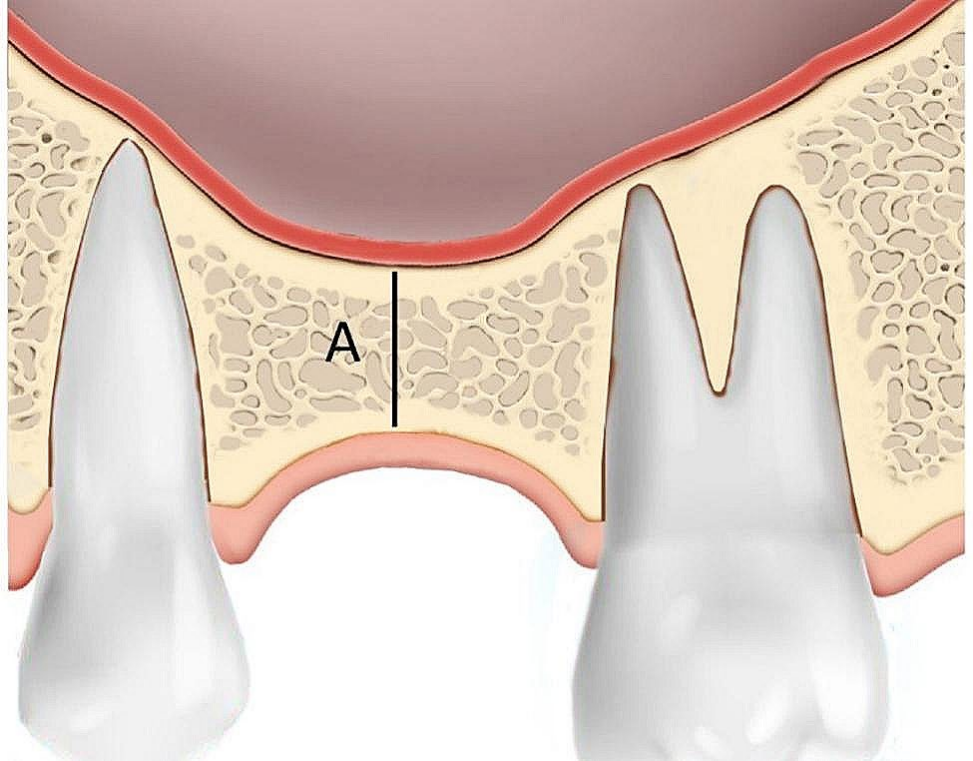
Residual bone height measurement (**A**); the shortest distance from the alveolar crest to the initial maxillary sinus floor on preoperative CBCT

**Fig. 3:**
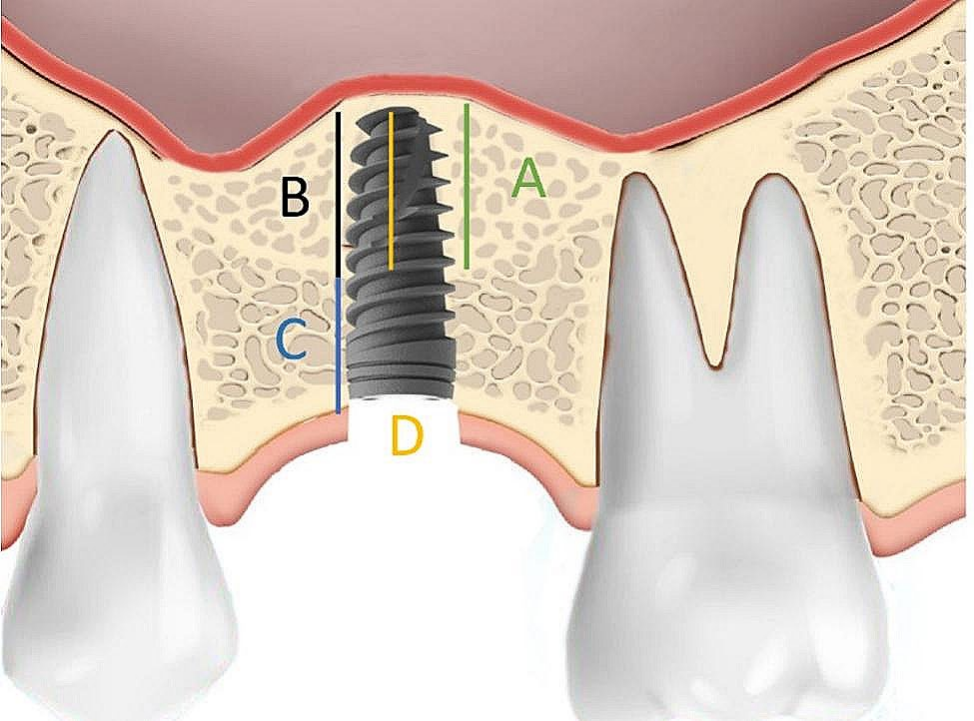
Postoperative measurement at one year after maxillary sinus lifting and implant insertion; vertical sinus floor elevation with implant (**A**); the distance between the initial sinus floor and the elevated sinus floor. Newly formed bone within the sinus cavity (**B**); the newly formed bone within the distance between the initial sinus floor and the elevated sinus floor. The total bone height after the sinus lifting (**B** + **C**); the sum of C and B. Implant protrusion (**D**); calculated as subtraction of implant length and C

**Fig. 4:**
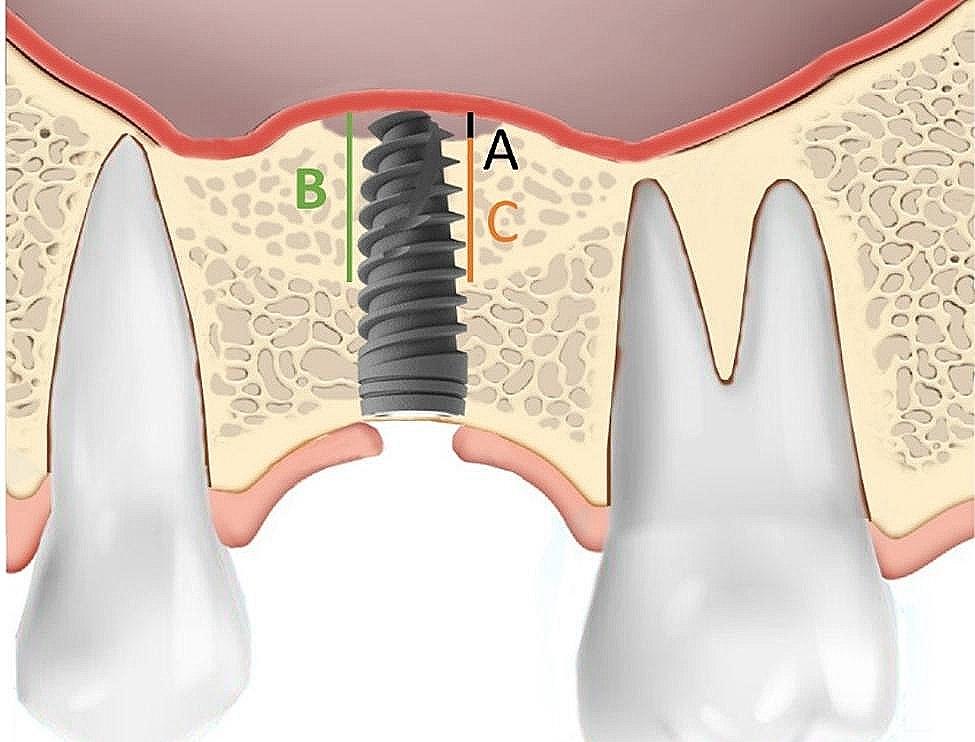
Postoperative measurement at one year after maxillary sinus lifting and implant insertion; vertical sinus floor elevation without new bone formation (**A**); calculated as subtraction of vertical sinus floor elevation with implant (**B**) and newly formed bone within the sinus cavity (**C**)

Invivo Dental 5.0 (Anatomage Inc., San Jose, CA, USA) was employed for radiographic assessment [9]. The specific location of implant placement and the dental implant itself served as reference points. The measurements of parameters are as follows (Figs. 2, 3 and 4; Table 1):

**Table 1:** Abbreviations and descriptions of parameters measurements analyzed

Abbreviation	Description	Parameters
RBH	Residual bone height	The shortest distance from the alveolar crest to the initial maxillary sinus floor on preoperative CBCT.
VSFE	Vertical sinus floor elevation with implant	The distance between the initial sinus floor and the elevated sinus floor.
IP	Implant protrusion	The distance between initial sinus floor and implant apex calculated as subtraction of implant length and RBH
NFBS	Newly formed bone within the sinus cavity	The newly formed bone within the distance between the initial sinus floor and the elevated sinus floor.
VSFE^− NBF^	Vertical sinus floor elevation without new bone formation	Calculated as subtraction of vertical sinus floor elevation with implant and newly formed bone within the sinus cavity
TBH	The total bone height at least one year after the sinus lifting	The sum of RBH and NFBS was measured at least one year after the sinus lifting operation
ISQ	The implant stability quotient (ISQ)	The mean values of occlusal and buccal ISQ.

i) Residual bone height (RBH): within estimated implant site, the shortest distance from the alveolar crest to the initial maxillary sinus floor on preoperative CBCT.
ii) Vertical sinus floor elevation with implant (VSFE): the distance between the initial sinus floor and the elevated sinus floor.
iii) Implant protrusion (IP): the distance between initial sinus floor and implant apex calculated as subtraction of implant length and RBH.
iv) Newly formed bone within the sinus cavity (NFBS); the newly formed bone within the distance between the initial sinus floor and the elevated sinus floor.
v) Vertical sinus floor elevation without new bone formation (VSFE^-NBF^); calculated as subtraction of VSFE and NFBS.
vi) The total bone height after the sinus lifting (TBH), calculated as the sum of RBH and NFBS was measured at least one year after the sinus lifting operation.

### Statistical analysis

Social Sciences (SPSS) version 27, (Chicago, USA) was proficiently employed for conducting various computations involving both descriptive and analytical statistics. To ensure the data’s normality distribution, the Kolmogorov-Smirnov test was employed. For intergroup comparisons, parametric data underwent analysis via the independent t-test. Moreover, the intraclass correlation coefficient test (ICC) was utilized to examine the intra-observer reliability of the measurements. The significance level was set at *P* < 0.05.

## Results

This clinical study was conducted on 60 sites in 60 patients who needed dental implants in the posterior maxilla and diagnosed with a reduced vertical bone height. The means age of A and B groups during maxillary sinus lifting were 42.7 ± 7.8 (27–59) & 43 ± 8.7 (27–55) years, postoperatively. There was no significant difference found among groups regarding gender, placement side, placement position, and follow up (Table 2).

**Table 2:** Demographic features of participants of groups

Demographic features	Freshly Extracted socket group	Healed socket group	*P*-Value
Gender, no. (%)			
Male Female	18 (60%) 12 (40%)	17 (56.6%) 13 (43.3%)	0.61
Placement Position, no. (%)			
Premolar area Molar area	4 (13.3%) 26 (86.7%)	6 (20%) 24 (80%)	0.17
Placement Side, no. (%)			
Right Left	23 (76.7%) 7 (23.3%)	24 (80%) 6 (20%)	0.38
Age, yrs			
(Mean ± S.D) (Min-Max)	42.7 ± 7.8 (27–59)	43 ± 8.7 (27–55)	0.22
Follow-up, yrs			
(Mean ± S.D) (Min-Max)	1.09 ± 0.1 (1-1.33)	1.11 ± 0.1 (1-1.33)	0.634

### Clinical outcomes

During the follow-up period, all dental implants of both groups (diameter of 4.5 and 10 mm length) showed 100% survival rate, with no biological or mechanical complications or failure, and the primary implant stability was satisfied, with no statistically significant difference (*P* = 0.38) between group A and group B (60.07 ± 8.83 & 55.10 ± 10.46) respectively **(**Table 3**)**.

**Table 3:** Inter-group comparisons using a millimeter measurement unit

Variables	Freshly extracted socket group	Healed socket group	*P*-Value
Residual bone height	6.88 ± 1.42	6.67 ± 1.56	0.68
Vertical sinus floor elevation with implant	3.12 ± 1.42	3.33 ± 1.56	0.68
Newly formed bone within the sinus cavity	3.34 ± 1.45	3.22 ± 1.18	0.26
Vertical sinus floor elevation without new bone formation*	+ 0.22 ± 0.33	-0.11 ± 0.64	0.30
The total bone height at least one year after the sinus lifting	10.22 ± 0.33	9.89 ± 0.64	0.03
The implant stability quotient (ISQ)	60.07 ± 8.83	55.10 ± 10.46	0.38

*Minus value (-) means the newly formed bone did no cover the whole implant height within the sinus cavity (implant protrusion), plus value (+) means the newly formed bone above the implant apex

### Radiographic outcomes

For all parameters, ICC tests were more than 0.96, indicating an acceptable level of agreement.

Regarding residual bone height, both groups showed no statistically significant difference (*P* = 0.68). Regarding height of sinus elevation with implant, both groups showed no statistically significant difference (*P* = 0.68), (group A; 3.12 ± 1.42 & group B; 3.33 ± 1.56).

Regarding the peri‑implant bone formation inside the sinus, both groups showed satisfied and successful outcomes in regard to bone gain around the implant with increased bone gain in group A, with no statistically significant difference (*P* = 0.26).

Regarding the difference between the dental implant height inside the sinus and the peri‑implant bone formation inside the sinus, group B showed 0.11 ± 0.64 mm of intrasinus implant height without bone formation, corresponding to the dental implant height of (3.33 ± 1.56) mm, while group A showed increment of bone formation above the intrasinus implant (0.22 ± 0.33) mm, corresponding to the dental implant height of (3.12 ± 1.42) mm, with no statistically significant difference between both groups (*P* = 0.30).

Regarding the total bone height, group A showed statistically more total bone height than group B (*P* = 0.03) (Table 3).

## Discussion

Minimally invasive surgeons consistently push boundaries, redefining what can be achieved through smaller incisions and significantly reducing surgical stress levels. Patients can enjoy several evident benefits from a minimally invasive surgical approach, including reduced postoperative pain, faster recovery, and economic savings resulting from a shorter recovery period.

**Fig. 5:**
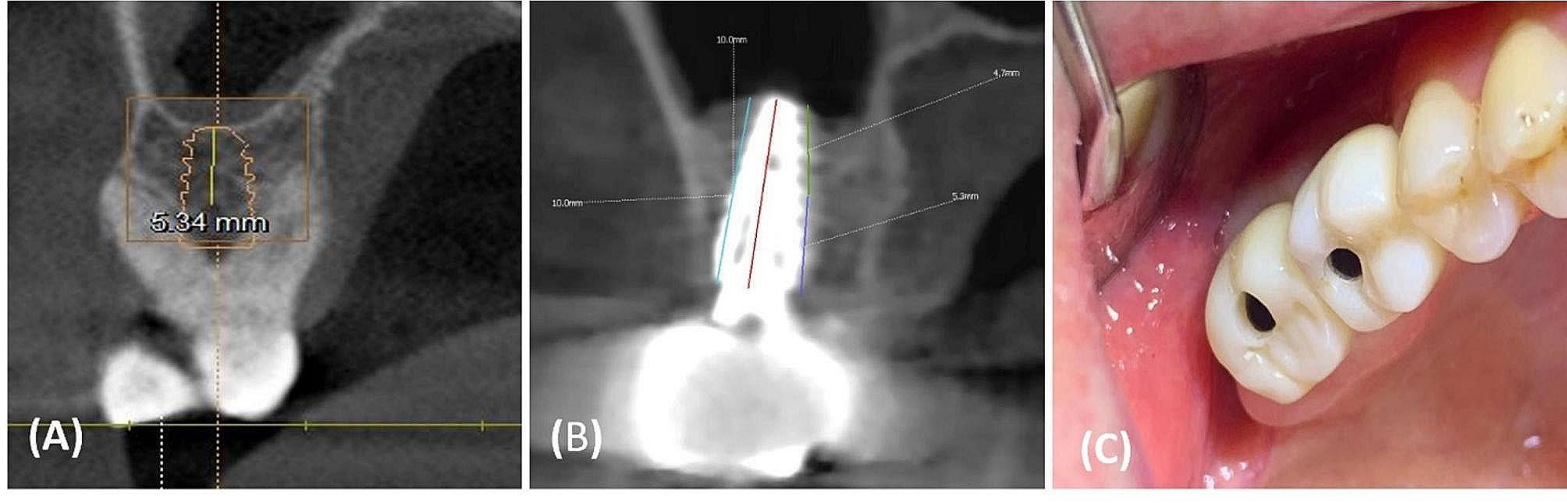
Preoperative and postoperative CBCT scan views of case with freshly extracted socket who needed dental implants in the posterior maxilla and was diagnosed with a reduced vertical bone height the maxillary sinus floor at right upper sex molar; (**A**) Preoperative coronal view of right upper sex molar, (**B**) Postoperative coronal view of right upper sex molar at one year after maxillary sinus lifting and implant insertion, (**C**) Postoperative clinical view

**Fig. 6:**
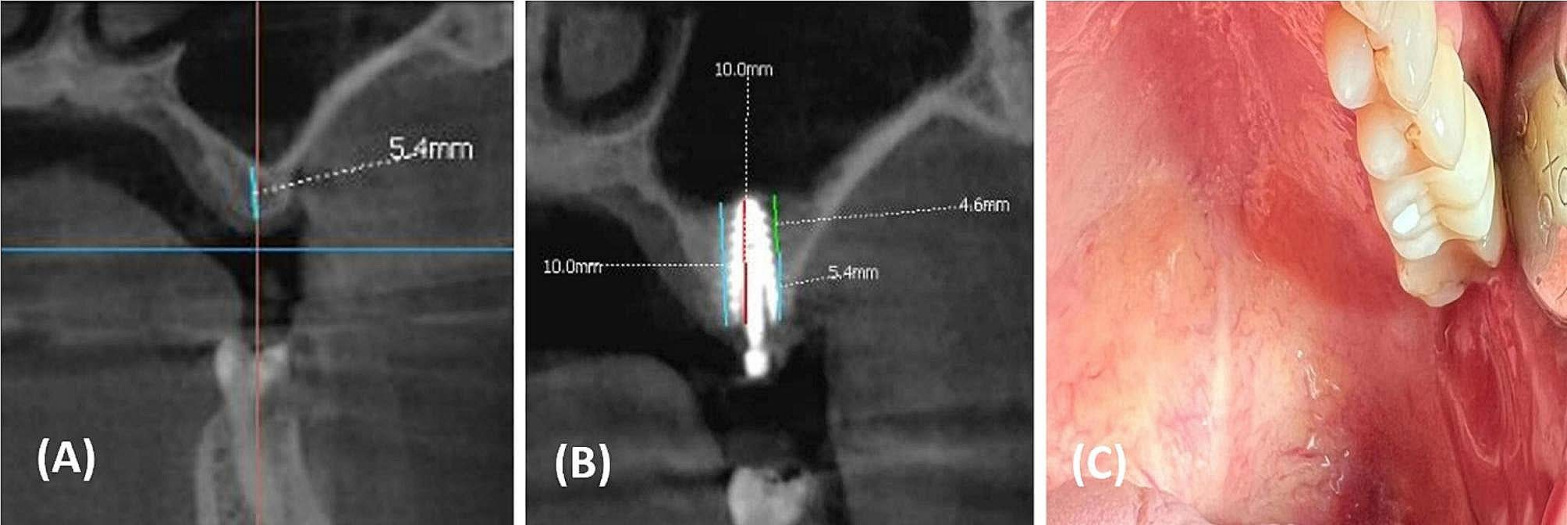
Preoperative and postoperative CBCT scan views of case with healed socket who needed dental implant in the posterior maxilla and was diagnosed with a reduced vertical bone height the maxillary sinus floor at left upper sex molar; (**A**) Preoperative coronal view of left upper sex molar, (**B**) Postoperative coronal view of left upper sex molar at one year after maxillary sinus lifting and implant insertion, (**C**) Postoperative clinical view

On the other hand, clinical studies have shown that new bone has the capability to develop naturally on and around implanted dental implants without the necessity of using bony substitutes. Nevertheless, the evident advantages in terms of cost-effectiveness and time-saving become apparent when implants are placed at the time of sinus lifting, left to osseointegrate without the need for autogenous bone or allografts [15, 21]. Thus, the aim of the present clinical study was to introduce a minimally graftless sinus lifting technique with simultaneous dental implant placement (Figs. 5 and 6). Based on the survival criteria proposed by Buser et al. [22], our results demonstrated a 100% survival rate for all dental implants in both groups, with no reported biological or mechanical complications. In a review of studies, Riben et al. [23] found that the graftless technique consistently exhibited high implant survival rates. Notably, this technique has proven to be cost-effective, significantly less time-consuming, and associated with lower morbidity. A systematic review on the survival of implants inserted using the transalveolar technique in combination with sinus floor elevation concluded that the rates of implant survival in transalveolar sinus floor augmentation sites are comparable to those in non-augmented sites [24]. On the other hand, the failure rate using the graftless technique is comparable to that of conventional techniques. Nevertheless, this technique offers the advantage of reduced contamination since it does not require external grafts or supplementary surgeries [25]. The stabilities of the implants placed into either fresh extraction sockets or at healed alveolar sites showed comparable evolutions in ISQ values at the three examined time intervals [26]. This finding aligns with our results; both groups exhibited satisfactory stability (Group A: 60.07 ± 8.83 and Group B: 55.10 ± 10.46) with no statistically significant difference between them (*P* = 0.38). Regarding residual bone height, all patients in both groups had presurgical residual bone heights exceeding 5 mm (6.88 ± 1.42 mm and 6.67 ± 1.56 mm), with no statistically significant difference (*P* = 0.68). This criterion aligns with studies by Summers et al. [11] and Rabah Nedir et al. [12]. The osteotome sinus floor elevation technique has consistently demonstrated reliable results in the posterior maxilla when the remaining bone height is above 5 mm.

Regarding intrasinus bone formation, a systematic review and meta-analysis reported a mean bone height gain of 3.43 ± 0.09 mm [27], which is consistent with our results (Group A: 3.34 ± 1.45 mm and Group B: 3.22 ± 1.18 mm, with a 100% survival rate). Rabah Nedir et a. [12]. confirmed that the graftless transalveolar sinus floor lifting procedure is sufficient to create bone beyond the natural limit of the sinus. In addition, at the three-year midpoint, it was concluded that the implants had acquired endo-sinus bone, and the bone gain had continued to improve since the first year. There is no need for exogenous graft materials, as the blood clot that developed underneath the lifted maxillary sinus membrane appears to be crucial for the possibility of bone neoformation [28–30]. Studies conducted both in vivo and in vitro have shed light on the mechanism and origin of osteoprogenitor cells that contribute to bone formation after graftless sinus elevation [31, 32]. On the other hand, Falah et al. [25] demonstrated that direct implant placement maintained a cavity for blood clot formation, while graftless bone neoformation occurred underneath the lifted maxillary sinus membrane. Additionally, the dental implant serves as the primary graft filler in the space between the sinus membrane and basal bone. Currently, Kadkhodazadeh et al. [26] evaluated the clinical and radiographic outcomes of the vertically expander screw (VES) technique, a novel graftless approach for maxillary sinus floor elevation and simultaneous implant placement. They concluded that the mean intrasinus bone gain using this approach was 5.44 ± 1.66 mm. This technique is consistent with our results regarding intrasinus bone gain; both groups demonstrated satisfactory and successful intrasinus bone formation (Group A: 3.34 ± 1.45 mm and Group B: 3.22 ± 1.18 mm) with no statistically significant difference (*P* = 0.26).

Regarding the mean difference between the sinus lifting height with the dental implant and the new bone formation inside the sinus, Group B showed 0.11 ± 0.64 mm of intrasinus implant without bone formation, corresponding to the dental implant height of (3.33 ± 1.56) mm. In contrast, Group A showed an increment of bone formation above the intrasinus implant (0.22 ± 0.33) mm, corresponding to the dental implant height of (3.12 ± 1.42) mm, with no statistically significant difference between the two groups (*P* = 0.30). The new bone formation was generally observed at the upper limit of the implant, and the quality of the clot formed directly influences the new bone formation [33]. On the other hand, stem cells, anchor elements, and growth factors play essential roles in the bone regeneration process. Therefore, the osteogenic potential of the sinus membrane and the bone next to the implant, serving as an anchor element, are key factors in the success of the technique [34]. Thus, the surgical procedure in a fresh socket may stimulate osteogenic activity and promote bone formation during the early stages of healing. In contrast, the bone in a healed socket has had time to undergo remodeling, which may influence the overall quality of the bone and, consequently, impact the success of the sinus lifting procedure.

Dental implants placed in fresh sockets present numerous benefits, including a reduction in overall treatment time and the number of surgical procedures [35]. Gehrke SA et al. [36] concluded that the immediate placement of implants can effectively prevent bone resorption and potentially lead to superior socket remodeling.

Overall, several transalveolar techniques for maxillary sinus elevation have been developed [13], including the expansion-based technique [26], drill-based technique [37], hydraulic pressure technique [38], piezoelectric surgery [39], and balloon technique [40]. The outcomes of these techniques align with the outcomes of our approach, demonstrating the superiority of our approach in terms of cost-effectiveness, time efficiency, and lower morbidity. Despite the limitations of this study, such as a small sample size, our approach appears to offer promising outcomes. Nevertheless, it is essential to conduct long-term histological studies to authenticate this technique and compare it with currently employed methods.

## Conclusion

Our approach demonstrated high survival rates with acceptable primary implant stability and satisfactory outcomes in terms of bone gain around the implant in both freshly extracted and healed sockets. Therefore, our approach holds promise as a procedure for sinus lifting with simultaneous dental implant placement.

## Data Availability

The datasets used and analyzed during the study are available from the corresponding author upon reasonable request.
